# Case Report: Delayed complications of endovascular treatment of recurrent erosive hemorrhage in necrotizing pancreatitis

**DOI:** 10.3389/fsurg.2026.1776415

**Published:** 2026-05-15

**Authors:** Mark Tokarev, Ivan Semenenko, Alina Zelenskaia, Baina Komiyukova, Viacheslav Shibitov

**Affiliations:** 1 University Clinic of General, Reconstructive and Cardiovascular Surgery, I.M. Sechenov First Moscow State Medical University of the Ministry of Health of Russia (Sechenov University), Moscow, Russia; 2 University Clinic of General, Reconstructive and Cardiovascular Surgery, Moscow State Budgetary Healthcare Institution “Moscow City Hospital Named After S.S. Yudin, Moscow Healthcare Department”, Moscow, Russia

**Keywords:** acute pancreatitis, embolization, endovascular treatment, intraabdominal hemorrhage, necrotizing pancreatitis, pancreatic necrosis, splenic abscess formation

## Abstract

In our case, NP was complicated by recurrent erosive hemorrhage from branches of the splenic artery, requiring multiple endovascular interventions, and by the development of a post-embolization splenic abscess. This case illustrates the complexity of treating vascular complications in pancreatic necrosis and the importance of monitoring for delayed complications of endovascular procedures. In addition to the well-recognized risk of hemorrhage in necrotizing pancreatitis, vascular interventions themselves may lead to delayed ischemic complications. The coexistence of recurrent arterial bleeding requiring stepwise embolization and subsequent splenic abscess formation represents a complex clinical scenario that illustrates the challenges of therapeutic decision-making in severe pancreatitis and remains insufficiently discussed in the literature.

## Introduction

Necrotizing pancreatitis (NP) is a severe form of acute pancreatitis characterized by enzymatic destruction of the pancreatic parenchyma and a pronounced systemic inflammatory response. The mortality rate in severe cases reaches 20%–30% (1), which is primarily due to the development of organ failure and secondary complications. These include: infected necrosis [which significantly increases mortality (2–4)], erosive hemorrhage (5), mechanical jaundice, duodenal obstruction, thrombosis of visceral vessels, pancreatic fistulas, and abdominal compartment syndrome (3, 4, 6).

The mortality rate associated with vascular complications in the setting of NP reaches 34%, especially when emergency laparotomy is performed early in the course of the disease (5). Currently, international guidelines and many authors consider endovascular hemostasis to be the preferred treatment method in hemodynamically stable patients (3–5, 7). However, such interventions may be accompanied by delayed complications: splenic infarction and splenic abscess (8). In the context of infected pancreatic necrosis, the risk of abscess development increases, since ischemic splenic tissue becomes particularly vulnerable to bacterial translocation (9). Recurrences of bleeding have also been described in cases of incomplete vessel occlusion, as well as ischemic changes in the stomach following embolization of the short gastric arteries (10, 11).

In our case, NP was complicated by recurrent erosive hemorrhage from branches of the splenic artery, requiring multiple endovascular interventions, and by the development of a post-embolization splenic abscess. This case illustrates the complexity of treating vascular complications in pancreatic necrosis and the importance of monitoring for delayed complications of endovascular procedures.

In addition to the well-recognized risk of hemorrhage in necrotizing pancreatitis, vascular interventions themselves may lead to delayed ischemic complications. The coexistence of recurrent arterial bleeding requiring stepwise embolization and subsequent splenic abscess formation represents a complex clinical scenario that illustrates the challenges of therapeutic decision-making in severe pancreatitis and remains insufficiently discussed in the literature.

## Case presentation

The patient, S., is a 46-year-old man. He was admitted with a clinical picture of severe acute pancreatitis: intense pain in the epigastric and mesogastric regions, nausea, and jaundiced discoloration of the skin and sclera, along with laboratory evidence of a systemic inflammatory response and organ dysfunction. Markedly elevated blood urea and creatinine levels indicated acute renal failure (2 points on the Marshall score), which defines severe acute pancreatitis according to the revised Atlanta classification (12).

**Figure 1:**
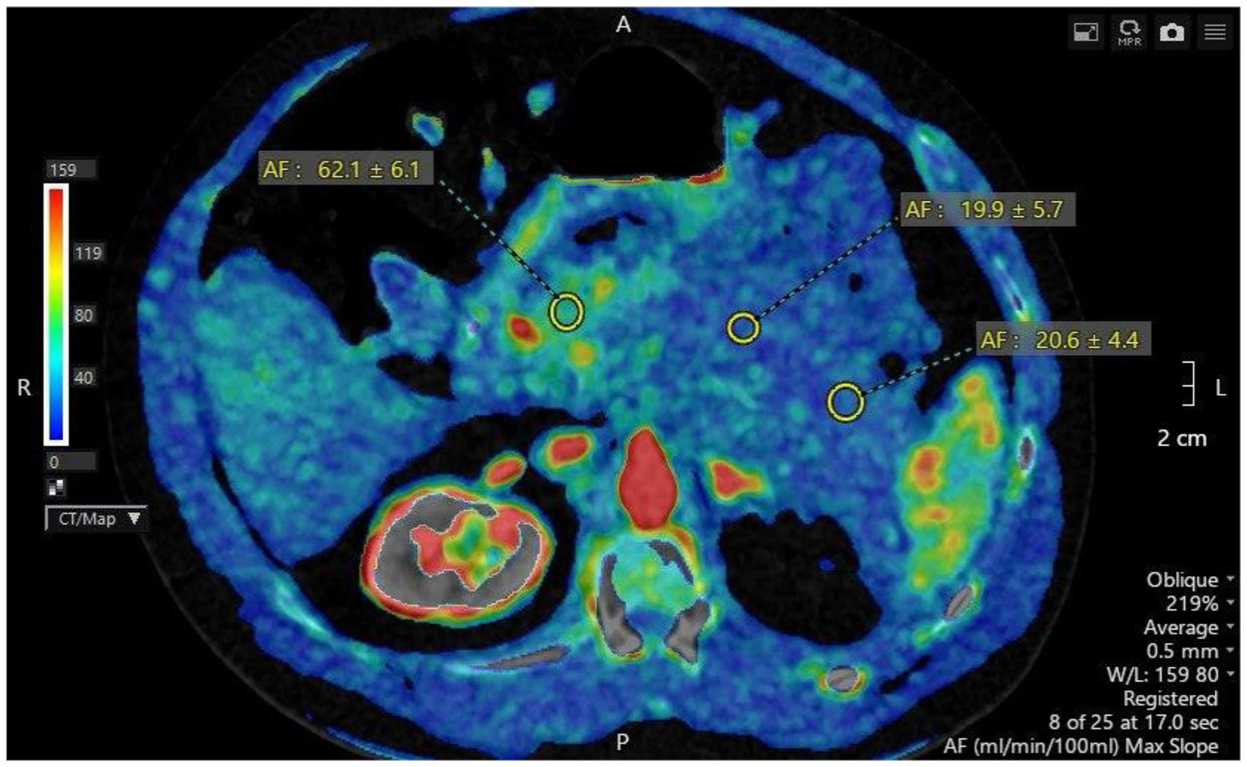
Perfusion CT of the pancreas.

Perfusion CT of the pancreas showed a moderate reduction of perfusion in the head (AF = 62.1 ± 6.1 mL/min/100 mL) and a pronounced reduction of perfusion in the body and tail (AF = 19.9 ± 5.7 and 20.6 ± 4.4 mL/min/100 mL, respectively) (13). These findings are shown in Figure 1.

On the 5th day of illness percutaneous drainage of the omental bursa and left retroperitoneal space was performed under ultrasound guidance. Although international guidelines generally recommend delaying invasive intervention in severe pancreatitis until necrotic collections become walled-off (4), early drainage may be considered in the presence of clinical deterioration, persistent systemic inflammatory response, or suspected infection. In the present case, given the rapidly progressing systemic inflammatory response and the absence of clinical improvement with conservative therapy, early image-guided percutaneous drainage was performed as part of a step-up approach. The intervention was limited to drainage alone, without necrosectomy, in order to control infection while maintaining the least invasive treatment strategy during the acute phase of the disease. The drainage fluid showed a high alpha-amylase activity (1,498 U/L), indicating the formation of an internal pancreatic fistula. Clinical improvement was observed: the signs of systemic inflammation subsided, and renal function parameters and bilirubin level normalized. The patient was discharged 12 days later with the drain in place.

**Figure 2:**
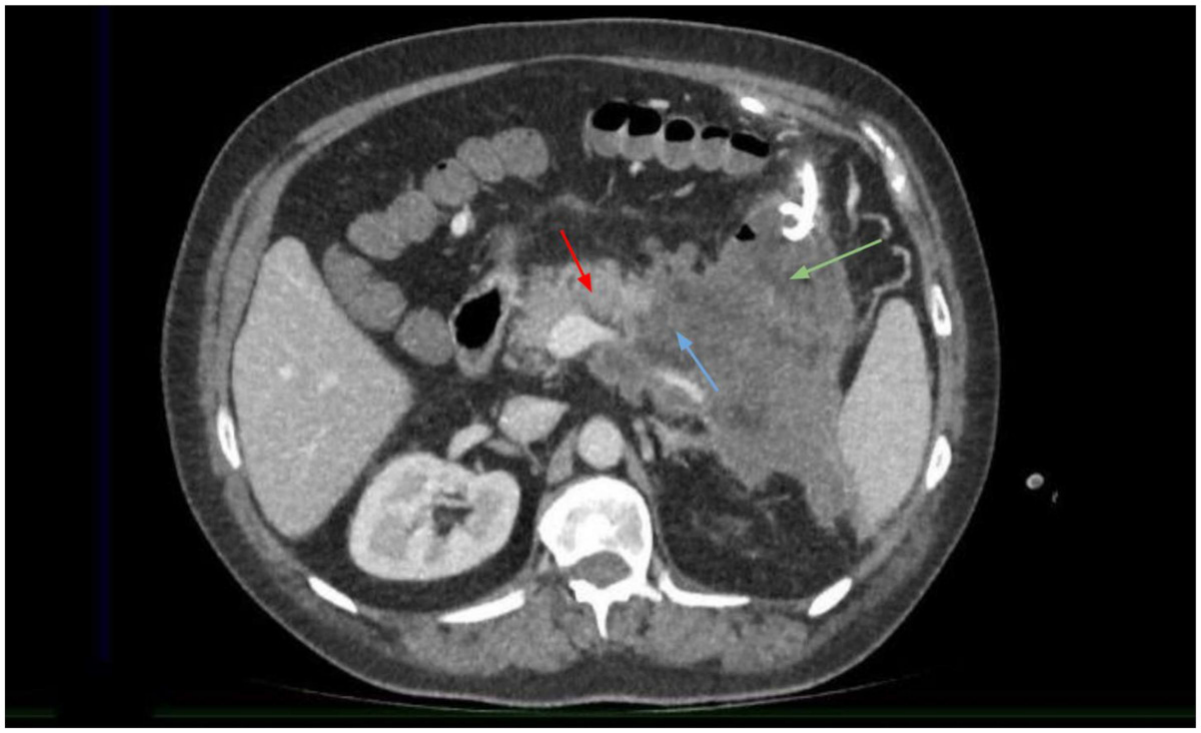
Contrast-enhanced CT of the abdomen: red arrow—viable pancreatic parenchyma; blue arrow—extensive necrosis of the pancreatic body and tail; green arrow—fluid collections within the omental Bursa.

Thirty days after symptom onset, the patient was readmitted with signs of systemic inflammatory reaction and abdominal pain. Drainage catheters in the epigastric and left iliac regions (placed as an outpatient procedure) yielded up to 50 mL of brown serous-fibrinous fluid. Contrast-enhanced abdominal CT revealed signs of NP: areas of deep necrosis of the pancreatic parenchyma in the body and tail; large accumulations of fluid and debris in the omental bursa and left retroperitoneal space (up to 10 × 6 cm) (14, 19). These findings are shown in Figure 2. Diffuse parapancreatitis was classified as Ishikawa Grade IV (14).

Considering the signs of infected necrosis and the ineffectiveness of percutaneous drainage, a retroperitoneoscopic necrosectomy was performed four weeks after symptom onset.

**Figure 3:**
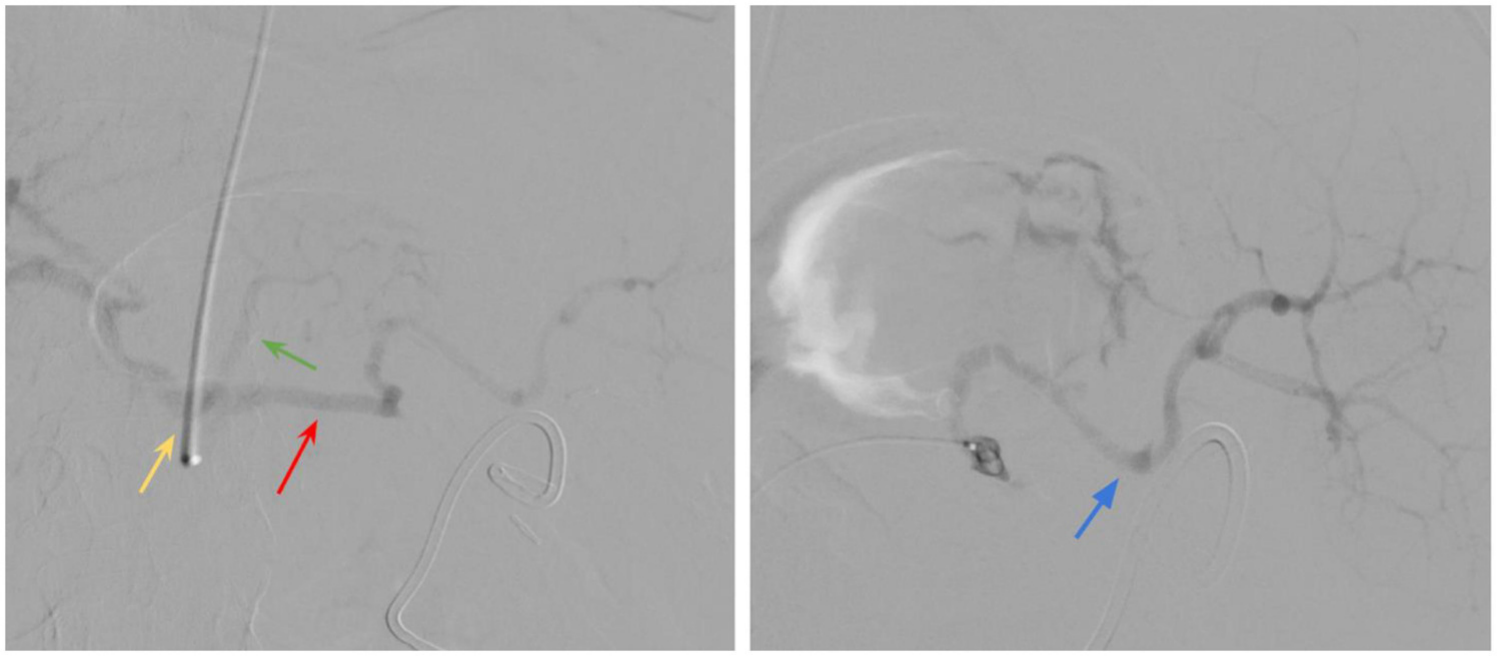
CT angiography. Catheterization of the celiac trunk (yellow arrow), left gastric artery (green arrow), splenic artery (red arrow), and splenic artery branch (blue arrow); no evidence of contrast extravasation.

Three days after the surgery, the drain placed near the pancreatic tail began to yield up to 100 mL of hemorrhagic fluid. Clinically, this was accompanied by hemodynamic instability and a decrease in hemoglobin to 9.3 g/dL. Urgent CT angiography was performed: the celiac trunk was catheterized, and a branch of the splenic artery in the pancreatic tail region was selectively embolized with microemboli to achieve hemostasis. Representative angiographic findings are shown in Figure 3.

Eleven days later, a recurrent intra-abdominal hemorrhage occurred: approximately 600 mL of hemorrhagic fluid was drained, again accompanied by hemodynamic instability. Repeat CT angiography was performed with selective microembolization of another splenic artery branch. However, the following day approximately 400 mL of serosanguinous discharge appeared, indicating a recurrence of bleeding. Despite the absence of clear contrast extravasation, given the clinical context and recurrent hemorrhage, proximal embolization of the splenic artery trunk was performed. No further episodes of bleeding occurred, and the patient was discharged in stable condition.

**Figure 4:**
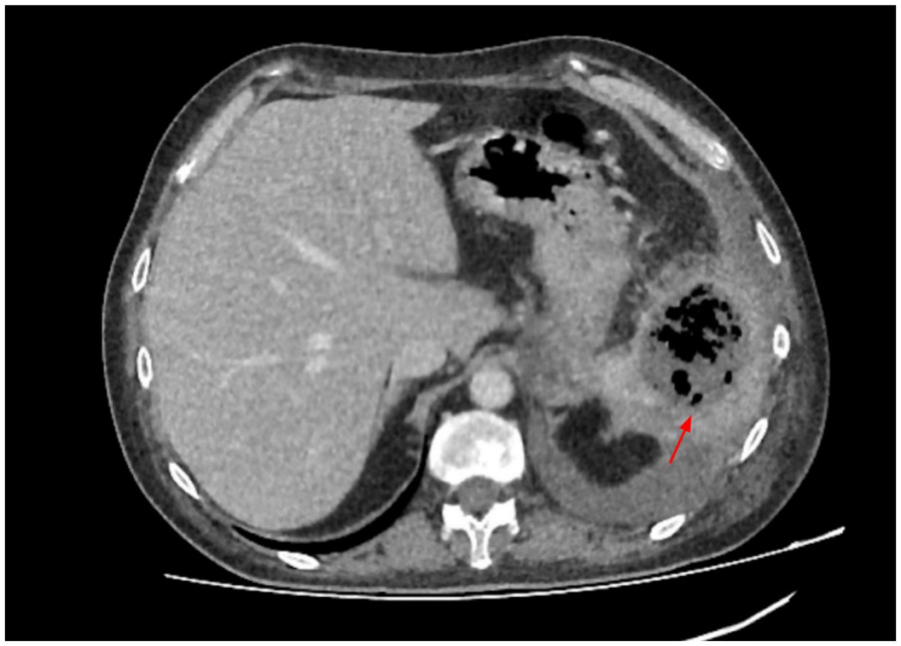
Contrast-enhanced CT of the abdomen. Red arrow—splenic abscess.

Three months later, the patient was readmitted with signs of systemic inflammation. Abdominal CT revealed a gas-containing fluid collection in the spleen region (splenic abscess) measuring approximately 7 × 6 cm. This finding is shown in Figure 4.

Ultrasound-guided percutaneous drainage of the abscess was performed, yielding pus; the cavity was irrigated. With continued antibiotic therapy and drainage, the patient's body temperature normalized and inflammatory markers decreased. The patient was discharged in satisfactory condition. The clinical course is summarized in Table 1.

**Table 1:** Clinical timeline.

Time from admission	Clinical event
Day 0	Hospital admission with severe acute pancreatitis
Day 5	Image-guided percutaneous drainage of pancreatic collections
Day 34	First episode of arterial hemorrhage
Day 34	Selective embolization of splenic artery branch
Days 45–46	Recurrent bleeding
Day 46	Proximal embolization of splenic artery trunk
Day 93	Development of splenic abscess
Day 94	Image-guided percutaneous drainage of splenic abscess

## Follow-up and outcomes

The patient demonstrated gradual clinical improvement following percutaneous drainage of the splenic abscess. Inflammatory markers progressively decreased and no further episodes of gastrointestinal bleeding occurred. Follow-up imaging demonstrated resolution of the abscess cavity; structural changes of the spleen consistent with post-procedural alterations without evidence of active hemorrhage. During a 3-month follow-up period, the patient remained clinically stable without recurrent hemorrhage or new infectious complications. No hematologic abnormalities suggestive of functional asplenia were observed.

## Literature review

Erosive hemorrhage and visceral artery pseudoaneurysms represent some of the most severe local complications of NP. The incidence of vascular complications in acute pancreatitis is reported to be approximately 1%–6% overall (5), increasing to 1.5%–8.5% in patients with severe pancreatitis (7). The splenic artery is the most frequent source of erosive hemorrhage (30%–50% of cases), while the gastroduodenal and pancreaticoduodenal arteries account for 20%–25% of cases (5). Bleeding in NP usually develops during the 3rd to 5th week of illness (5, 7).

International guidelines and numerous authors emphasize that, in hemodynamically stable patients, endovascular embolization is the preferred method of hemostasis, whereas laparotomy (splenectomy or vessel ligation) is reserved for cases in which endovascular treatment is ineffective or unavailable (3, 4, 7).

In a cohort of 105 patients with vascular complications of pancreatitis, endovascular treatment demonstrated high efficacy (5):

- technical success—98%;
- clinical success—93.2%;
- rebleeding rate—6.8%;
- procedure-related mortality—approximately 1%.

Similar results have been reported by other invstigators: in patients with severe acute pancreatitis and erosive hemorrhage, embolization achieved successful hemostasis in 96.3% of cases (7). A meta-analysis confirmed that endovascular embolization of pseudoaneurysms in pancreatitis provides high technical success (∼95%–97%) and clinical success of about 80%–90%, with rebleeding rates of 5%–15% (15).

In some patients, despite a clinical picture of hemorrhage, angiography may fail to reveal contrast extravasation. In such cases, proximal embolization of the suspected source vessel is performed based on CT findings (necrosis location) and indirect angiographic signs (vessel spasm, irregular vessel wall contour, pseudoaneurysm formation) (5).

However, splenic artery embolization (SAE) is associated with several delayed complications. Reviews and meta-analyses report that, in the general population undergoing SAE for blunt non-penetrating splenic trauma, the incidence of splenic abscess is approximately 1%–4% while splenic infarction occurs in 3%–6% of patients (8, 16, 17). The risk of complications depends on the extent and technique of embolization (proximal, distal, or combined). Nevertheless, SAE in trauma setting is generally considered a spleen-preserving intervention with a high success rate.

The situation changes significantly when embolization is performed in the context of infected pancreatic necrosis. The ischemic spleen lies adjacent to infected peripancreatic fluid collections and necrotic tissue, facilitating bacterial translocation. In a study of patients with severe acute pancreatitis who underwent SAE for massive hemorrhage, the incidence of splenic abscess formation was high (18 of 20 patients), and embolization itself was identified as a major contributing factor in the development of this complication (9).

Most patients with severe acute pancreatitis had the splenic abscess diagnosed early (within the first week after embolization) (9). However, cases of delayed abscess formation have also been reported—6–8 weeks and even 4 months after embolization—underscoring the importance of long-term follow-up in such patients (18).

Organ-preserving methods are preferred in the management of post-embolization splenic abscesses. Li et al. demonstrated that, in patients with infected pancreatic necrosis complicated by splenic abscess after SAE, complete resolution was achieved in 56% of cases (10 of 18 patients) using ultrasound-guided percutaneous drainage (9). Quencer et al. emphasize that drainage is the treatment of choice in hemodynamically stable patients, whereas splenectomy is indicated only when minimally invasive therapy fails or when CT demonstrates absent perfusion, a septated abscess, total splenic necrosis, or signs of progressive sepsis (16).

## Discussion

This clinical case illustrates a severe vascular complication of necrotizing pancreatitis—erosive hemorrhage—which results from the enzymatic destruction of the arterial wall by necrotic processes. The clinical picture (hemodynamic instability, hemorrhagic drainage output, and a decrease in hemoglobin levels) required urgent intervention. In the setting of infected pancreatic necrosis, endovascular intervention allowed us to avoid laparotomy, which is associated with significantly higher mortality rates (5). The present case illustrates the complexity of treatment decision-making in necrotizing pancreatitis complicated by recurrent hemorrhage from the splenic artery basin.

The hemorrhage developed on day 34 after symptom onset, which is consistent with published data indicating that hemorrhagic complications in necrotizing pancreatitis typically occur during the 3rd to 5th week of illness (5, 7). The bleeding originated from branches of the splenic artery, which is characteristic in cases with necrosis of the pancreatic tail.

In the present case, selective embolization of the bleeding splenic artery branches was initially preferred in order to preserve splenic perfusion and reduce the risk of ischemic complications. Selective embolization allows targeted hemostasis while maintaining collateral arterial supply and is generally considered the first-line endovascular approach when the bleeding source can be clearly identified. However, despite two initial selective embolization procedures, the patient developed recurrent clinically significant hemorrhage accompanied by clinical deterioration. Under these circumstances, proximal embolization of the splenic artery trunk was performed as a salvage strategy to reduce arterial inflow pressure and achieve durable hemostasis.

Alternative management strategies for hemorrhage associated with necrotizing pancreatitis include surgical intervention such as splenectomy or combined surgical-endovascular approaches. However, surgery during the early inflammatory phase of severe pancreatitis is associated with substantial morbidity due to tissue friability and ongoing systemic inflammation. For this reason, endovascular treatment is currently considered the preferred first-line approach for arterial bleeding in pancreatitis. In the present case, surgical intervention was reserved as a potential rescue option in the event of persistent hemorrhage or failure of endovascular therapy.

Splenic infarction and abscess formation are recognized complications after splenic artery embolization, particularly when proximal embolization or repeated embolization procedures are required. The risk may be increased in the setting of severe pancreatic necrosis and persistent peripancreatic inflammation, which can compromise collateral perfusion and promote secondary infection. In the present case, the combination of extensive necrotizing pancreatitis and repeated embolization likely contributed to the development of delayed splenic abscess.

Although angiography did not demonstrate overt contrast extravasation, subsequent proximal embolization of the splenic artery trunk was performed and successfully achieved durable hemostasis.

Proximal embolization of the splenic artery led to splenic ischemia, and adjacent infected fluid collections and necrotic areas created favorable conditions for bacterial translocation and subsequent splenic abscess formation. In our case, the abscess developed three months after embolization, emphasizing the need for long-term monitoring of such patients.

The management strategy for the abscess was determined by the patient's stable condition and the limited extent of the lesion. In accordance with the step-up approach principles, percutaneous drainage was preferred, which aligns with data demonstrating the efficacy of organ-preserving treatment (9, 16).

This case underlines the necessity of a cautious choice of embolization level in patients with infected necrotizing pancreatitis. Proximal or combined splenic artery occlusion provides reliable hemostasis but significantly increases the risk of ischemic complications. Even with successful cessation of bleeding, such patients require prolonged follow-up with routine ultrasound and/or CT monitoring. Organ-preserving methods can effectively manage post-embolization splenic abscesses and help avoid splenectomy, but they require timely recognition of complications.

Thus, our clinical observation highlights the importance of an individualized approach and a stepwise treatment strategy: from timely embolization to the management of delayed complications. A rational combination of endovascular and minimally invasive interventions helps minimize the risk of adverse outcomes in patients with severe necrotizing pancreatitis.

## Conclusion

This clinical case illustrates a scenario characteristic of severe acute pancreatitis, in which successful resolution of one complication may predispose to the development of another. Sequential endovascular interventions ensured reliable hemostasis, but in the setting of infected pancreatic necrosis increased the risk of post-embolization abscess formation. This underscores the need to assess the consequences of the chosen treatment strategy and to provide long-term monitoring of patients. Early recognition of ischemic and purulent-septic complications of embolization, combined with timely and minimally invasive management, enables safe continuation of treatment in patients with severe pancreatic necrosis.

## Patient perspective

The patient reported gradual recovery following hospital discharge and expressed satisfaction with the minimally invasive treatment strategy, particularly avoidance of major surgical intervention during the acute phase of illness. He provided written informed consent for publication of this case and hopes that sharing his experience will help clinicians manage similar cases in the future.

## Data Availability

The datasets presented in this article are not readily available because access is restricted due to patient confidentiality and institutional policies. Only anonymized, non-identifiable data may be shared upon reasonable request. Requests to access the datasets should be directed to Alina Zelenskaia, zelenskaia.alina@gmail.com.
